# Risk Assessment of Nanomaterials Toxicity

**DOI:** 10.3390/nano13091512

**Published:** 2023-04-28

**Authors:** Andrea Hartwig, Christoph van Thriel

**Affiliations:** 1 Department of Food Chemistry and Toxicology, Institute of Applied Biosciences (IAB), Karlsruhe Institute of Technology (KIT), Gebäude 50.41 (AVG), Adenauerring 20a, 76131 Karlsruhe, Germany; andrea.hartwig@kit.edu; 2 Leibniz Research Centre for Working Environment and Human Factors, Research Group Neurotoxicology and Chemosensation, Department of Toxicology, TU Dortmund, Ardey Str. 67, 44139 Dortmund, Germany

The increasing use of nanomaterials in almost every area of our daily life renders toxicological risk assessment a major requirement for their safe handling. Thus, risk assessment strategies ensuring the health of individuals exposed to these types of materials must be adopted and continuously reviewed. Major challenges include the enormous amount of engineered nanomaterials (ENMs) used in workplaces [1], the limited capacity for testing ENMs in long-term animal inhalation studies [2], and the political and societal efforts to reduce animal experiments according to the 3R principles [3]. Against this background, much attention has been paid to grouping of nanomaterials, mainly based on their physicochemical properties and their toxicity in various in vitro models. These new approach methodologies (NAMs) include a detailed characterization of the respective materials in physiologically relevant media, but also more realistic exposure systems, such as co-cultures, at the air–liquid interface, combined with comprehensive cellular investigations providing quite detailed toxicological profiles. These NAM-based approaches have been recently reviewed by the U.S. Federal Agencies and the authors concluded that “… two key issues in the usage of NAMs, namely dosimetry and interference/bias controls, …” are crucial aspects in ongoing validation processes [4]. In workplaces where inhalation is the major route of exposure, potential toxicity affecting the lungs needs to be considered. Here, advanced in vitro models have documented their predictive capacity for adverse outcomes such as lung fibrosis [5]. Neurotoxicity associated with exposure to nanomaterials is another growing field of scientific investigation [6] and, here, the use of nanocarriers for drug delivery provides a special “route of exposure” [7]. We initiated this Special Issue to further promote scientific progress in the area of nanosafety and are glad to share 13 papers on various topics with the readership of *Nanomaterials*. This Special Issue highlights recent advances in the mechanisms of nanomaterial toxicity as well as approaches for risk assessment, linking nanoparticle characteristics and in vitro toxicity to in vivo observations for advanced risk assessment. Here, the availability of data and the development of databases are important.

With three original articles by Murugadoss, Mülhopt et al., Elje et al., and Meindl et al., addressing various aspects, the respiratory tract toxicities of titanium dioxide, carbon nanotubes, and nanosilver have been described and some assays can be further validated. A link between in vitro screening and results from in vivo testing for lung effects is provided by Creutzenberg et al., describing results from the PLATOX project. Focusing on the aspect of data availability and reproducibility, Krug describes the development of the CoCoN-Database, while Elberskirch et al. describe the results of a round-robin test that includes data science tools to increase comparability among different labs. Another relevant and important aspect is addressed by de Souza Castro et al., comparing 2D and 3D cell culture models of bone mineralization. Here, the 3D model showed improved induction of bone osteointegration by nanoparticles. Mechanisms related to the possible genotoxicity of ENM are described in the papers by Schumacher et al., May et al., and Murugadoss, with Godderis et al. also addressing the crucial aspect of realistic exposure scenarios in vitro. These papers are also relevant to the key issue of dosimetry, as described by Petersen et al. [4]. The paper by Wall et al. provides new insight into the physico-chemical properties of particulate and fibrous nanomaterials that can modulate their toxicity. Finally, the review by Ruijter et al. highlights various aspects of how in vitro methods can be incorporated into the *Safe-by-Design* concept that is expected to foster the development of safe ENMs before they enter the market.
